# Purity matters: A workflow for the valid high-resolution lipid profiling of mitochondria from cell culture samples

**DOI:** 10.1038/srep21107

**Published:** 2016-02-19

**Authors:** Lisa Kappler, Jia Li, Hans-Ulrich Häring, Cora Weigert, Rainer Lehmann, Guowang Xu, Miriam Hoene

**Affiliations:** 1 Division of Clinical Chemistry and Pathobiochemistry, Department of Diagnostic Laboratory Medicine, University Hospital Tuebingen, Tuebingen, Germany; 2 Key Laboratory of Separation Science for Analytical Chemistry, Dalian Institute of Chemical Physics, Chinese Academy of Sciences, Dalian, China; 3 Department of Molecular Diabetology, Institute for Diabetes Research and Metabolic Diseases of the Helmholtz Centre Munich at the University of Tuebingen, Tuebingen, Germany; 4 German Center for Diabetes Research (DZD), Tuebingen, Germany

## Abstract

Subcellular lipidomics is a novel field of research that requires the careful combination of several pre-analytical and analytical steps. To define a reliable strategy for mitochondrial lipid profiling, we performed a systematic comparison of different mitochondria isolation procedures by western blot analyses and comprehensive high-resolution lipidomics. Using liver-derived HepG2 cells, we compared three common mitochondria isolation methods, differential centrifugation (DC), ultracentrifugation (UC) and a magnetic bead-assisted method (MACS). In total, 397 lipid species, including 32 cardiolipins, could be quantified in only 100 μg (by protein) of purified mitochondria. Mitochondria isolated by UC showed the highest enrichment in the mitochondria-specific cardiolipins as well as their precursors, phosphatidylglycerols. Mitochondrial fractions obtained by the commonly used DC and the more recent MACS method contained substantial contaminations by other organelles. Employing these isolation methods when performing lipidomics analyses from cell culture mitochondria may lead to inaccurate results. To conclude, we present a protocol how to obtain reliable mitochondria-specific lipid profiles from cell culture samples and show that quality controls are indispensable when performing mitochondria lipidomics.

Mitochondria are coming more and more into the focus of basic and translational research due to their central metabolic function and their involvement in the pathophysiology of human diseases such as neurodegenerative disorders, cancer and diabetes^1^,^2^,^3^. Mitochondria have an essential role in the regulation of bioenergetic processes, cell stress response and epigenetic modulations^4^,^5^,^6^. Besides β-oxidation, the main pathway for fatty acid oxidation^7^, mitochondria are also sites of lipid synthesis and remodelling^8^. Mitochondria synthesize approximately 45% of their own phospholipids^9^, mostly phosphatidylethanolamines (PE), phosphatidylglycerols (PG), phosphatidic acids (PA) and cardiolipins (CL)^8^,^10^. Lipids are not simply membrane constituents, but also involved in physiological processes such as mitochondrial fusion and fission, membrane structure and fluidity, electron transport chain assemblage, protein biogenesis, apoptosis and many more^11^,^12^,^13^,^14^,^15^,^16^. The role of lipids in mitochondrial function is exemplified by CL, the mitochondrial signature phospholipid, which has been shown to be a key player in the organization of the general mitochondrial membrane structure and in the organization of the essential electron transport chain components into higher order assemblies^12^,^15^,^17^,^18^. An accurate, comprehensive lipid profiling strategy is therefore a prerequisite to investigate the contribution of individual lipids to mitochondrial (dys-) function. However, most lipids are not specific for individual organelles and therefore challenging to quantify in a mitochondria-specific fashion. Thus, an isolation procedure which enriches mitochondria and minimizes contaminations by membranes from other organelles is a major prerequisite for the reliable profiling of mitochondrial lipids.

The existence of diverse mitochondria isolation procedures with different requirements regarding time and equipment and basing on different principles of separation makes a decision for the most suitable method quite challenging for a scientist planning to perform “omics” analyses. Impurities caused by contaminations with other organelles may not only result in misleading analytical findings, but also hamper the comparability of data from different groups^15^,^19^. We therefore aimed to establish a valid, robust and sensitive workflow for the isolation of mitochondria from cell culture samples and subsequent lipid profiling by ultra performance liquid chromatography (UPLC)-LTQ-Orbitrap-mass spectrometry (MS). To this end, we systematically compared three different isolation procedures and the composition and characteristics of the resulting lipid profiles, focussing on the purity of the yielded mitochondrial fraction. We compared two commonly used methods, differential centrifugation (DC)^20^,^21^,^22^,^23^ and DC followed by ultracentrifugation (UC) on a Percoll gradient^21^,^23^,^24^, with a more recent magnetic bead-assisted method (MACS)^25^,^26^. Using liver-derived HepG2 cells, we defined a strategy for the valid, comprehensive and reproducible investigation of the lipidome of pure mitochondria based on as little as 100 μg (by protein) of isolated sample material.

## Results and Discussion

Lipidomics analyses covering cardiolipins have previously been performed using liquid chromatography (LC)-MS and less often shotgun^11^, gas chromatography (GC)-MS^22^ or MALDI-TOF-MS techniques on mitochondria from various tissues^20^,^21^,^23^,^27^,^28^, macrophages and yeast^28^,^29^. In addition to the different analytical approaches, lipidomics data published so far originated from very different mitochondria isolation methods: Either DC^20^,^21^,^22^,^23^ or UC on density gradients like Iodixanol^29^, Ficoll and Sucrose^11^,^27^, or Percoll^21^,^23^,^24^.

**Figure 1:**
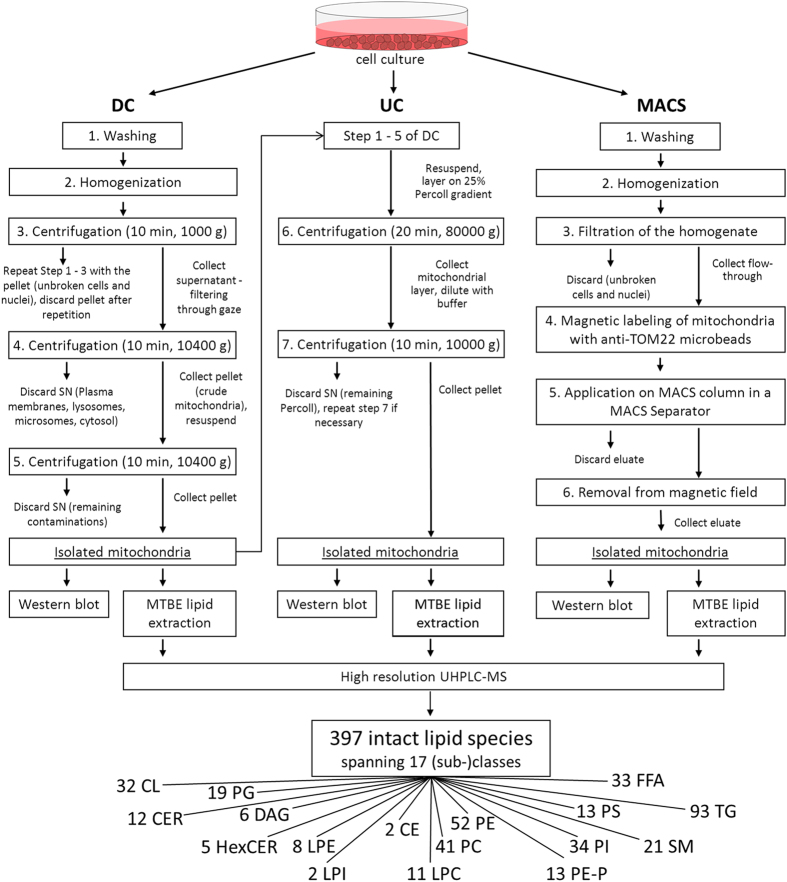
Experimental workflow from sample preparation to western blot and lipidomics analyses. Three mitochondria isolation methods, differential centrifugation (DC), ultracentrifugation (UC) and magnetic bead-assisted isolation (MACS) were systematically compared; see Methods section for further details. SN, supernatant; MTBE, methyl tert-butyl ether; CE, cholesteryl ester; CER, ceramide; CL, cardiolipin; DAG, diacylglycerol; FFA, free fatty acids; HexCER, hexosyl-ceramide; LPC, lyso-phosphatidylcholine; LPE, lyso-phosphatidylethanolamine; LPI, lyso-phosphatidylinositol; PC, phosphatidylcholine; PE, phosphatidylethanolamine; PE-P, phosphatidylethanolamine plasmalogen; PG, phosphatidylglycerol; PI, phosphatidylinositol; PS, phosphatidylserine; SM, sphingomyelin; TAG, triacylglycerol.

DC is the most simple method and frequently used since it is relatively fast and provides intact, functional mitochondria well suited to investigate mitochondrial physiology^30^,^31^. Briefly, tissues or cells are minced and homogenized, commonly using a Dounce homogenizer^28^,^32^,^33^. A slow centrifugation step is performed to sediment broken cells and cell debris, which is followed by a fast centrifugation step using the supernatant to pellet the mitochondria (Fig. 1, left). A higher purity can be achieved by subsequent high-speed centrifugation on a density gradient (Fig. 1, middle). This additional step can, however, reduce the yield of mitochondrial protein. An additional limiting factor is the necessity to utilize an ultracentrifuge, which may not be accessible for every scientist. In addition, centrifugation-based methods tend to be time-consuming, particularly when numerous samples are isolated simultaneously. Thus, a more rapid approach to isolate mitochondria has been commercialized that was first described by Hornig-Do *et al.*^26^. After sample homogenization, mitochondria are coupled to anti-TOM22-antibodies conjugated to paramagnetic beads. Bead-coupled mitochondria can then be isolated in a magnetic field (Fig. 1, right). This so-called MACS procedure has higher yields^26^ and liver mitochondria isolated by MACS have been shown to exhibit higher oxygen consumption rates than mitochondria isolated by DC^25^. To our knowledge, a magnetic bead-based mitochondria isolation strategy has not been used for lipidomics analyses so far.

### Organelle-specific protein abundances of mitochondria isolated by different purification methods

As a first step, we systematically compared mitochondria isolated from HepG2 cells by three different isolation methods, DC, UC, and MACS (Fig. 1) on the protein level. Each purification procedure was performed in at least five replicates and compared to total cell lysate.

**Figure 2:**
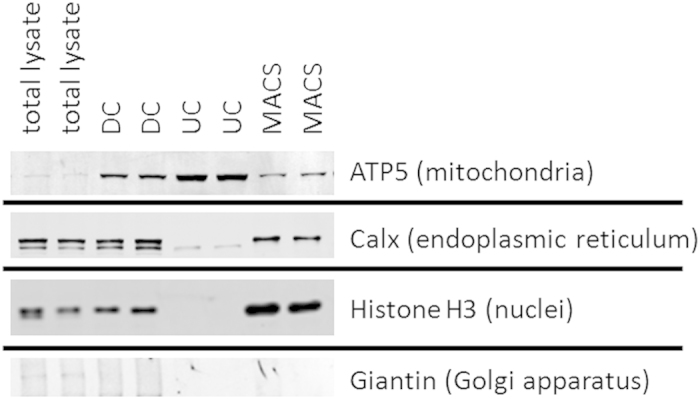
Analysis of organelle-specific proteins in total HepG2 cell lysates and in mitochondrial fractions obtained by differential centrifugation (DC), ultracentrifugation (UC) and magnetic bead-assisted isolation (MACS). A representative western blot of 2 mitochondria isolations per method is shown. Mitochondrial membrane ATP synthase 5 (ATP5) was used as a marker for mitochondria. For the detection of potential contaminations, antibodies against Calnexin (Calx), Histone H3 and Giantin were used.

By employing western blot analysis, organelle-specific markers for mitochondria as well as markers for potential contaminations by endoplasmic reticulum (ER), nuclei and Golgi apparatus (Fig. 2) as well as lipid droplets were investigated. With the exception of the nuclear marker histone, all antibodies were directed against membrane proteins. Equal protein amounts (30 μg) of total cell lysates were assessed as controls to determine the organelle-specific protein levels before enrichment. Mitochondrial membrane ATP synthase 5 (ATP5) was used to confirm the presence of mitochondria in total cell lysates and their enrichment in the isolated fractions (Fig. 2). UC samples showed the strongest ATP5 signal, indicating the most pronounced enrichment in mitochondria. At the same time, the markers for ER and nuclei were most efficiently depleted by UC. The lipid droplet marker PLIN2 was detectable in 90 μg of total HepG2 lysate (data not shown), but not in 30 μg of total lysate and mitochondrial fractions, indicating no enrichment of lipid droplets. In contrast, DC and in particular MACS samples contained prominent amounts of the markers for ER and nuclei (Fig. 2). All mitochondria isolation procedures led to a decrease of Giantin, the Golgi apparatus marker, compared to total cell lysates.

### Comparative lipid profiling of mitochondria isolated by different purification methods

In total, 397 lipids from 17 (sub-) classes, 12 ceramide (CER), 32 cardiolipin (CL), 2 cholesteryl ester (CE), 6 diacylglycerol (DAG), 5 hexosyl-ceramide (HexCER), 11 lyso-phosphatidylcholine (LPC), 8 lyso-phosphatidylethanolamine (LPE), 2 lyso-phosphatidylinositol (LPI), 41 phosphatidylcholine (PC), 52 phosphatidylethanolamine (PE), 13 phosphatidylethanolamine plasmalogen (PE-P), 19 phosphatidylglycerol (PG), 34 phosphatidylinositol (PI), 13 phosphatidylserine (PS), 21 sphingomyelin (SM), 93 triacylglycerol (TAG) and 33 free fatty acid (FFA) species could be quantified (Supplementary Table 1). We obtained a linear range over 2.0–3.2 orders of magnitude (r^2^ > 0.991) for the 5 CL standards tested. About 80% of all detected lipid species showed a relative standard deviation (RSD) below 30%, the RSD median value was 21.49% for the 6 different biological replicates obtained by UC purification. Of note, these deviations include all variable factors from mitochondrial isolation, lipid extraction and UPLC-MS analysis, showing high reproducibility and reliability of the whole (pre-) analytical procedure. The different isolation methods had no major effect on the total number of detected lipid species, but UC purification caused a depletion of 6 of the 93 TAG species to levels below the detection limit. The small sample amount employed, 100 μg (by protein) of purified mitochondria, did not cause a reduction in the number of detected lipid species compared to total cell lysate, but 3 of the 32 cardiolipins were below the detection limit in total cell lysate. The yield of mitochondria is often the bottleneck when it comes to a limited sample amount available, e.g. small tissue samples or samples with a low mitochondrial density. So far, published lipidomics analyses covering CLs required and used 250 μg^23^ to 1.5 mg^20^,^21^ (by protein) of mitochondria. Despite using as little as 100 μg, our lipidomics analysis was sufficiently sensitive to detect 32 cardiolipin species, whereas previous publications reported a detection of 27 species^20^,^27^ or less^20^,^29^.

**Figure 3:**
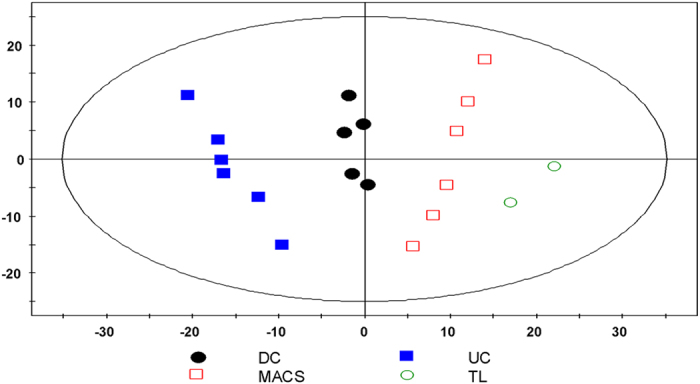
Principal Component Analysis (PCA) score plot. Each spot represents one mitochondrial sample isolated from HepG2 cells using three different methods: DC, differential centrifugation; UC, ultracentrifugation; MACS, magnetic bead-assisted isolation. TL, total cell lysate. All lipid variables with relative standard deviation less than 20% in the quality control samples were used for PCA modeling. Values were pretreated by unit variance (uv) scaling. Shown are the first two principal components (PC1 = 0.41, PC2 = 0.21); R^2^X = 0.865, Q^2^ = 0.748.

To gain an initial overview of the effect of the isolation procedures on the lipid profiles, a multivariate principal component analysis (PCA, Fig. 3) of the lipid compositions of the different mitochondrial samples and of total cell lysate was performed. In the direction of the first principal component a pronounced separation of the mitochondrial samples, which clustered together dependent on the isolation methods, could be seen. The difference from total cell lysate was smallest for MACS samples, followed by DC and UC. Thus, MACS isolation yielded mitochondrial samples whose lipid profile was closest to the profile of total cell lysate, whereas lipids from UC-isolated mitochondria were most distinct from total cell lysate.

### Mitochondrial samples isolated from HepG2 cells by UC show the highest enrichment in the mitochondria-specific cardiolipins

**Figure 4:**
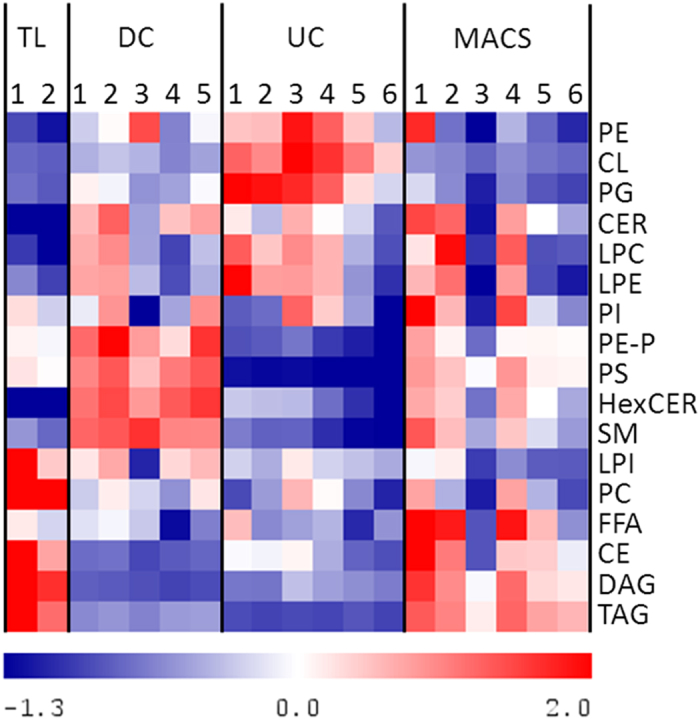
Heatmap visualization of the different lipid (sub-) classes in total cell lysates (TL) and in mitochondrial samples isolated by three different methods: Differential centrifugation (DC), ultracentrifugation (UC), and magnetic bead-assisted isolation (MACS). Each column represents an individual mitochondrial isolation. Values were centred to the mean of the respective lipid class and scaled to unit variance. White colour shows values close to the mean of the lipid class and red- and blue- coloured values are higher and lower, respectively, than the mean. CE, cholesteryl ester; CER, ceramide; CL, cardiolipin; DAG, diacylglycerol; FFA, free fatty acids; HexCER, hexosyl-ceramide; LPC, lyso-phosphatidylcholine; LPE, lyso-phosphatidylethanolamine; LPI, lyso-phosphatidylinositol; PC, phosphatidylcholine; PE, phosphatidylethanolamine; PE-P, phosphatidylethanolamine plasmalogen; PG, phosphatidylglycerol; PI, phosphatidylinositol; PS, phosphatidylserine; SM, sphingomyelin; TAG, triacylglycerol. The mitochondria-specific CLs were most enriched by UC.

To assess how the isolation methods affected the lipid composition of the samples, a heatmap was generated using the summed amounts of the individual lipid classes (Fig. 4). Data were normalized by mean centring and scaled to unit variance. In accordance with the PCA analysis, the heatmap revealed that MACS samples were most similar to total cell lysate, both showing higher levels of FFA, CE, DAG and TAG and lower levels of CL and PG than the other isolation methods. While DC samples contained the highest amounts of PE-P, PS, HexCER and SM, UC samples showed the greatest enrichment of PE, PG and CL.

Compared to other lipid classes, the abundance of CL, a mitochondria-specific dimeric glycerophospholipid, is relatively low in whole cell lysates^34^. While containing similar amounts of total lipids per μg of mitochondrial protein analysed, the different isolation methods yielded significantly different CL levels (Table 1). UC-purified mitochondria contained the highest relative amount (12.9 ± 1.7% of all phospholipids), DC was intermediate (3.6 ± 0.5% of all phospholipids) and MACS showed the lowest amount of CL (2.4 ± 0.3% of all phospholipids) (p_UC-DC_ < 0.0001, p_UC-MACS_ < 0.0001). In addition to CL, mitochondria also synthesize its precursor PG^14^. Thus, the elevated PG levels in UC samples further underpinned the higher mitochondria enrichment by UC compared to the other isolation methods.

While PE shared the lipid pattern observable for CL and PG, it is an abundant phospholipid with little specificity for mitochondria. However, a high PE/PC ratio is characteristic for mitochondria as a remnant of their bacterial background^9^. Interestingly, the lipids in the UC fraction exhibited the highest PE/PC ratio, 0.98 ± 0.08, followed by DC with 0.79 ± 0.19 and MACS with 0.66 ± 0.17, providing additional evidence that UC yielded the purest mitochondrial fraction.

### The lipid composition of mitochondrial samples isolated from HepG2 cells by DC and MACS supports a relevant contamination with membranes from other organelles

**Figure 5:**
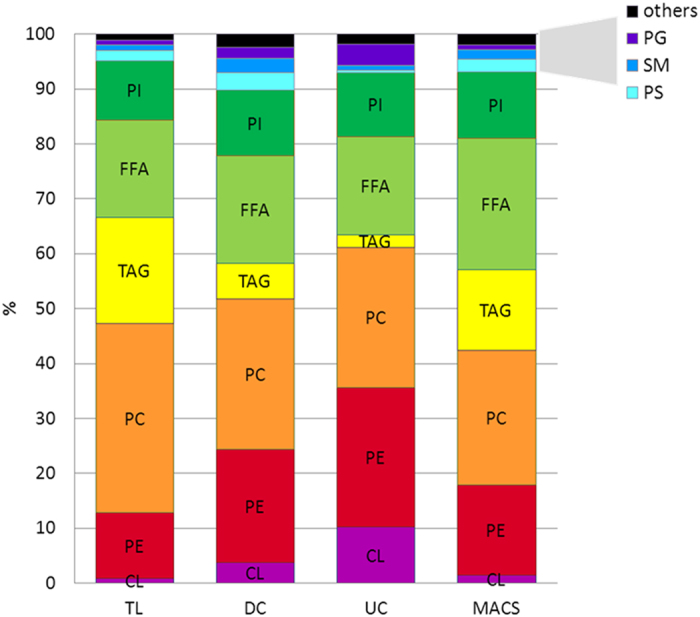
Contribution of the different lipid classes to all lipids detected in mitochondria enriched by differential centrifugation (DC), ultracentrifugation (UC) or magnetic bead-assisted isolation (MACS) and in total cell lysates (TL). Values are means of at least 5 purifications or of 2 total cell lysates. CL, cardiolipin; FFA, free fatty acids; PC, phosphatidylcholine; PE, phosphatidylethanolamine; PG, phosphatidylglycerol; PI, phosphatidylinositol; PS, phosphatidylserine; SM, sphingomyelin; TAG, triacylglycerol.

Western blot analyses indicated that in addition to enriching the mitochondria-specific protein ATP5, DC and MACS also enriched markers of other organelles (Fig. 2). UC-purified samples, in contrast, depleted Histone H3 beyond detectability and only a very low signal was visible for the ER-marker Calnexin (Fig. 2). In a similar manner, DC and MACS also increased the relative amounts of other lipid classes that were not enriched by UC (Fig. 5). Compared to UC, samples isolated by DC or MACS contained significantly higher amounts of PS, PE-P, SM, and HexCER (Table 1). Sphingolipids are characteristically high in ER, lysosomes, Golgi apparatus and plasma membranes^14^,^35^. As the mitochondrial sphingolipid content is normally very low, high levels of SM and HexCER in mitochondrial fractions can likely be attributed to contaminations by other organelles and membranes. Similarly, the PS content has been reported to be higher in ER, Golgi, lysosomes and the plasma membrane than in mitochondria^14^. Contaminations could result from co-sedimentation (mainly during DC), from organelle-mitochondria interactions, or from non-specific interactions with the antibody or the solid phase during the MACS procedure. PE plasmalogens (PE-P) were significantly lower with UC than with the other isolation methods. Mitochondria from heart, brain, kidney, adrenal cortex and spleen are rich in PC and PE plasmalogens^14^,^35^. While the liver is the major site for plasmalogen synthesis^36^, PE-P are present only at low levels in liver mitochondria^37^,^38^,^39^. Since plasmalogens also make part of nuclear, ER and Golgi membranes^37^,^38^, the higher PE-P in the DC- and MACS-samples might indicate contaminations by these other organelles.

A striking feature of the MACS and, to a lower degree, DC samples was their significantly higher TAG content (Figs 4 and 5 and Table 1). While TAGs are mainly stored in lipid droplets, neutral lipid cores surrounded by a monolayer of phospholipids and associated proteins^40^, the ER is the major site of their production^41^, pointing towards a relevant contamination of the samples isolated by MACS and DC with ER membranes and/or lipid droplets.

**Figure 6:**
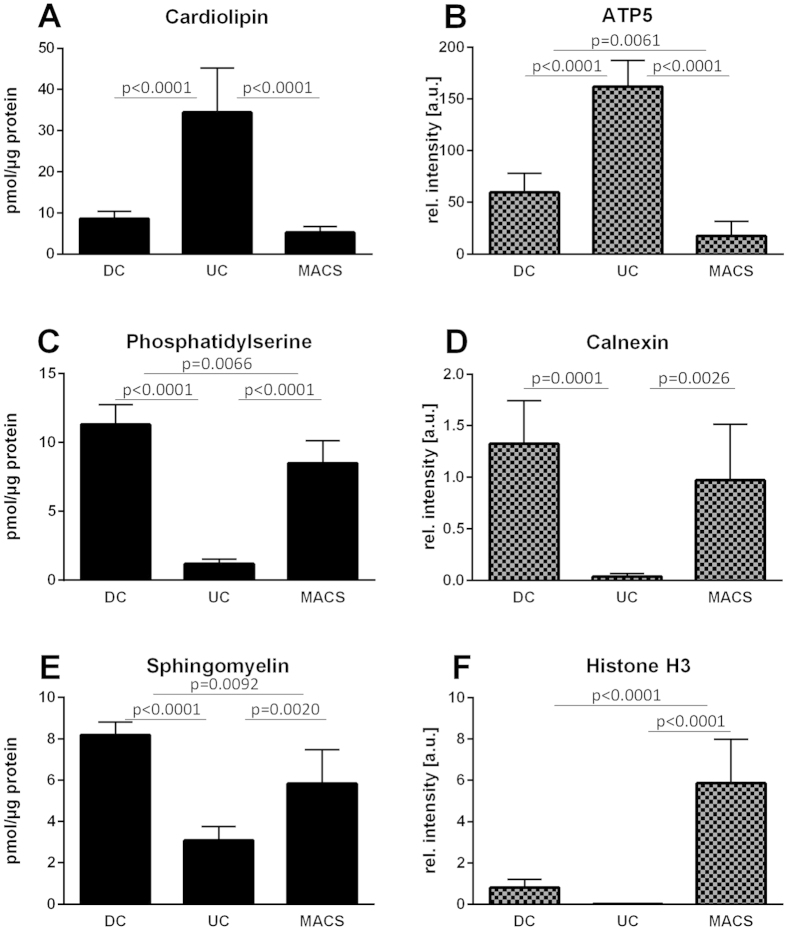
Comparison of lipid (left) and protein contents (right) in mitochondrial samples isolated using three different procedures, differential centrifugation (DC), ultracentrifugation (UC), and magnetic bead-assisted isolation (MACS). Histograms show the sums of the lipid classes or the densitometric quantifications of western blots normalized to total cell lysate. Values are means ± SD of at least 5 purifications.

To assess whether lipidomics data alone can provide an estimate of the purity of mitochondrial samples, we performed a direct comparison of lipidomics and western blot data (Fig. 6). Both CL, the signature lipid of mitochondria, and ATP5 were pronouncedly enriched in UC samples, resulting in a strong correlation between CL concentrations and ATP5 protein levels in the purified mitochondria (r^2^=0.79, p < 0.0001; Supplementary Figure 1; Fig. 6A,B). This is in line with previous observations in skeletal muscle tissue that suggested CL to be a superior marker of mitochondrial content^42^.

On the other hand, two lipid classes, PS and SM, and the ER-specific protein Calnexin were reduced in UC-purified samples in a similar fashion (Fig. 6C–E). Since lipids other than CL cannot be assigned to individual organelles, protein analyses are better suited to classify residual ER or other contaminants in mitochondrial samples. Nevertheless, the higher sensitivity and the possibility to investigate contaminations in a quantitative fashion make lipidomics an appropriate tool to assess the purity of mitochondrial samples.

Our results clearly show that UC, but not DC or MACS, yielded a pure mitochondrial fraction with little contamination by non-mitochondrial organelles. Different from functional assays, which highly depend on mitochondrial integrity and viability, the quality of subcellular “omics” analyses is directly related to sample purity. Tests for contaminations or quality controls of mitochondrial enrichment are rarely reported. However, lipidomics studies performed on DC- and MACS-isolated mitochondria may result in erroneous conclusions if the samples contain relevant amounts of ER and other organelles, as indicated in our study. In addition, the application of different protocols with different degrees of contaminations might cause an incomparability of the results from different studies. For example, published CL contents range between 10 to 20%^14^,^43^ of total phospholipids, which has been suggested to be caused not solely by different experimental conditions, but also by varying degrees of purity of the mitochondrial samples^15^. Therefore, we strongly recommend purity examinations by western blot or comparable assays in the early phase of an experiment in order to ensure the quality and reliability of publications involving lipidomics analyses of mitochondrial samples.

## Conclusion

Mitochondria isolation by ultracentrifugation yielded the purest samples and is therefore strongly recommended for lipidomics analyses of mitochondria obtained from cell culture models. The two other methods tested, DC and MACS, may result in misleading conclusions when isolating mitochondria for ”omics” analyses. The lipidomics data obtained in this study confirmed CL to be a potent marker for mitochondrial content, comparable or even superior to purity controls by western blot analysis. In addition, we showed that elevated amounts of PS, HexCER, SM, and TAG may indicate contaminations by other organelles. Thus, a careful analysis of lipid profiles can be recommended as quality control of mitochondrial samples in analytical settings. Subcellular profiling is becoming increasingly relevant for basic and translational research. The implementation of valid, robust sample preparation strategies is one of the keys to success for these sophisticated studies. Although DC and MACS are much faster and easier to perform, we highly recommend purification by UC to achieve reliable results when performing mitochondrial lipid profiling.

## Experimental Procedures

### Chemicals and Materials

RPMI medium 1640 was purchased from Gibco, life technologies (Carlsbad, CA, USA). Fetal bovine serum (FBS) was from Biochrom GmbH (Berlin, Germany). Trypsin and penicillin/streptomycin were from Lonza Ltd (Basel, Switzerland). HepG2 cells were purchased from DSMZ (Braunschweig, Germany). The human mitochondria isolation kit was purchased from Miltenyi Biotec (Bergisch Gladbach, Germany). Bradford Reagent was from Carl Roth (Karlsruhe, Germany). Infrared fluorescent dye secondary antibodies (anti-mouse/-rabbit/-guinea pig) were purchased from LI-COR (Lincoln, NE, USA). Anti-Calnexin was from Santa Cruz Biotechnology Inc. (Dallas, TX, USA). Anti-Histone H3, anti-Giantin and anti-ATP5A antibodies were purchased from Abcam (Cambridge, UK). Anti-PLIN2 was purchased from Progen Biotechnik (Heidelberg, Germany). Bovine Serum Albumin (BSA) was from Sigma-Aldrich (St. Louis, MO, USA). Internal standards were purchased from Avanti Polar Lipids, Inc. (Alabaster, AL, USA), Sigma-Aldrich (Munich, Germany) and ten Brink (Amsterdam, The Netherlands). MS or LC grade solvents acetonitrile (ACN), methanol (MeOH) and isopropanol (IPA) were purchased from Merck (Darmstadt, Germany). Ammonium acetate was purchased from Sigma-Aldrich (St. Louis, MO, USA). Ultra-pure water was prepared by a Milli-Q system (Millipore, MA, USA). The synthetic lipid standards d4-palmitic acid, ceramide CER(d18:1/17:0), LPC(19:0), PC(19:0/19:0), PE(15:0/15:0), SM(d18:1/12:0), TAG(15:0/15:0/15:0), CL(14:0(4)), CL(24:1(3)-14:1), CL(14:1(3)-15:1), CL(15:0(3)-16:1) and CL(22:1(3)-14:1) were purchased from Avanti Polar Lipids (Alabaster, AL, USA) or Sigma-Aldrich (Taufkirchen, Germany).

### Methods

#### Cell culture

HepG2 cells were grown at 37 °C in RPMI 1640 medium with 10% FBS, 1% penicillin/streptomycin and 1% glutamine. Cells were seeded at 4 × 10^6^ in 15 cm cell culture plates and cultured for 3 days.

#### Mitochondria isolation procedures

For DC and UC (see Fig. 1) six 15 cm plates (approximately 90% confluent) were washed twice with ice-cold PBS, scraped and pooled into ice-cold STE buffer (250 mM Sucrose, 5 mM Tris, 2 mM EGTA, pH 7.4 at 4 °C) containing 0.5% BSA. Small aliquots of this suspension were removed and stored at −80 °C for total cell lysates and the remainder was used for mitochondria isolation. From now on all steps were performed at 4 °C or on ice. Cells were collected by centrifugation at 500 g for 5 min, resuspended in 20 ml ice-cold STE + BSA, homogenized using a loose fitted 40 ml glass-glass Dounce homogeniser (Wheaton, Millville, NJ, USA) by applying 15 strokes, and centrifuged at 1,000 g for 10 min. The supernatant was transferred into a new centrifuge tube by filtering through 250 μM gauze. The pellet which possibly contained unbroken cells was resuspended in 15 ml fresh STE + BSA, homogenised again with 15 strokes and centrifuged at 1,000 g for 10 min. This supernatant was also transferred into a centrifuge tube by filtering through 250 μM gauze. The supernatants were combined and centrifuged at 10,400 g for 10 min to pellet the crude mitochondrial fraction. The pellet was resuspended in STE and centrifuged again at 10,400 g for 10 min. The crude mitochondrial pellet was carefully resuspended in 100 μl STE and transferred into a 2 ml reaction vessel. After centrifugation at 16,000 g for 2 min, the supernatant was removed and the DC pellets were frozen at −80 °C or used further for UC. For UC, the pellets were resuspended in 200 μl STE, layered on 5 ml Percoll gradient (25%) and centrifuged for 20 min at 80,000 g. The lower of the two appearing layers was collected with a glass Pasteur pipette and transferred into a new centrifuge tube, filled up with STE and centrifuged 10 min at 10,000 g. The pellet was resuspended in a small volume of STE buffer. After centrifugation at 16,000 g for 2 min the supernatant was removed and the UC mitochondrial pellet was frozen at −80 °C. For MACS, the mitochondrial isolation was performed following the manufacturer’s instructions with some adaptions (see Fig. 1). Cells were detached by scraping and 1 × 10^7 ^cells (1.5 15 cm plates, approximately 90% confluent) were counted and used for one mitochondrial isolation. Cell lysis was performed as recommended in the manual and by the company’s technical support using a 27G 3/4 needle and syringe (15 strokes). Prior to the magnetic separation step, the sample was filtered through 70 μM gauze to avoid clogging of the column. After elution of the mitochondrial fraction and centrifugation at 16,000 g for 2 min, the pellet was resuspended in 100 μl of STE. After another centrifugation step, the supernatant was removed again and the pellet was frozen at −80 °C.

#### Quantification of mitochondrial protein

For protein determination and further downstream analyses, frozen sample pellets were resuspended in a small volume of water. Mitochondrial suspensions were diluted 1:200 in Bradford reagent. Absorbance was measured at 590 nm using a microplate reader and sample concentration was assessed using a BSA standard.

#### Lipid extraction

Lipids were extracted with methyl tert-butyl ether (MTBE) as described previously^44^ with slight modifications. Briefly, 100 μg (by protein) of mitochondrial or total lysate were filled up with water to a total volume of 100 μl and 350 μl of ice-cold methanol including internal standards were added. Samples were briefly vortexed, 1 ml of MTBE was added and the samples were shaken for 30 min at room temperature. After adding 250 μl of water and incubation at room temperature for 10 min, samples were centrifuged for 20 min at 1,000 g and 4 °C to induce phase separation.

#### Electrophoresis and western blot analysis

30 μg of protein from total lysate or isolated mitochondrial fraction were separated by SDS-PAGE (12.5% or 5–12.5% gradient). Proteins were transferred to PVDF membranes by semi-dry electroblotting. After incubation with the respective primary antibodies, bands were visualised with the appropriate secondary infrared antibodies and band intensity was measured using an Odyssey Infrared Imaging System (LI-COR, Lincoln, NE, USA).

#### Lipidomics analysis

Lipidomics profiling was performed using UPLC-LTQ-Orbitrap-MS as previously described^45^. A Waters ACQUITY UPLC system equipped with a C_8_ ACQUITY column (2.1 mm × 100 mm × 1.7 μm) (Milford, MA, USA) was applied for lipid separation. The elution solvents were A (ACN:H_2_O = 60:40) and B (IPA:ACN = 90:10), both containing 10 mM ammonium acetate. The initial elution was set at 32% B for the first 1.5 min, followed by linear increase to 85% B during the next 14 min. The solvent B was further increased to 97% during the next 0.1 min and maintained for 2.4 min, then equilibrated at 32% for 2 min for the next injection. The column temperature was set to 55 °C and the flow rate was 0.26 ml/min. Lipidomics data were acquired in both ESI positive and negative modes at a mass resolution of 30,000 with scan ranges of 400–2000 and 90–2000, respectively. The positive mode was operated at a source voltage of 4 kV, capillary temperature of 300 °C and sheath gas and auxiliary gas flows of 25 arb and 5 arb. The respective parameter settings in the negative mode were 5 kV, 325 °C, 45 arb and 8 arb. Lipid identities were assigned based on accurate mass measurement, MS/MS fragmentation and elution behaviour. All detected lipids were quantified by normalization to the corresponding internal standard. The lipid nomenclature follows the LIPID MAPS classification and nomenclature system^46^. With our high-resolution UPLC-MS approach, a total of 401 lipids were detected in a pooled sample from DC-, UC-, MACS- purified mitochondria and total cell lysate. About 99% of all detected lipids showed a RSD below 20% in four injections of the pooled sample during the 8-hour UPLC-MS run. After applying the 80% rule^47^ and removing lipids with >20% RSD, 397 lipids were quantified for further comparative analysis.

#### Statistical analysis

Data are presented as means ± standard deviation (SD). Statistical significance was evaluated by one-way ANOVA and Tukey’s post hoc test to account for multiple comparisons using GraphPad Prism (GraphPad Software Inc., La Jolla, CA, USA). A p-value < 0.05 was considered significant. Regression analysis was performed with the statistical software package JMP 11.0.0 (SAS Institute, Cary, NC, USA). Multivariate principal component analysis (PCA) was performed with SIMCA-P 11.5 (Umetrics AB, Umeå, Sweden). The open-source MultiExperiment Viewer software^48^ was employed for heatmap generation using mean centred data pre-scaled to unit variance (uv).

## Additional Information

**How to cite this article**: Kappler, L. *et al.* Purity matters: A workflow for the valid high-resolution lipid profiling of mitochondria from cell culture samples. *Sci. Rep.* **6**, 21107; doi: 10.1038/srep21107 (2016).

## Supplementary Material

Supplementary Table 1

Supplementary Information

## Figures and Tables

**Table 1:** Summed content of lipid classes quantified by LC-MS lipid profiling of mitochondria purified using different isolation methods (pmol/μg protein).

Lipid(sub-)class	DC		UC		MACS		UC vs. DC	UC vs. MACS	DC vs. MACS
	Mean	SD	Mean	SD	Mean	SD	*p-*value	*p-*value	*p-*value
CE	0.012	0.001	0.019	0.007	0.033	0.017	n.s.	n.s.	0.0135
CER	2.838	0.404	2.447	0.326	2.703	0.662	n.s.	n.s.	n.s.
CL	8.623	1.779	34.433	10.814	5.217	1.522	<0.0001	<0.0001	n.s.
DAG	0.092	0.007	0.129	0.018	0.283	0.077	n.s.	0.0002	<0.0001
FFA	59.601	10.134	60.664	11.180	87.242	28.114	n.s.	n.s.	n.s.
HexCER	1.013	0.076	0.516	0.113	0.719	0.151	<0.0001	0.0282	0.0033
LPC	1.625	0.459	1.834	0.476	1.709	0.727	n.s.	n.s.	n.s.
LPE	0.728	0.163	0.838	0.251	0.699	0.249	n.s.	n.s.	n.s.
LPI	0.081	0.032	0.064	0.010	0.052	0.021	n.s.	n.s.	n.s.
PC	91.406	5.802	86.358	11.261	89.657	14.345	n.s.	n.s.	n.s.
PE	72.193	17.171	85.393	16.767	60.249	24.064	n.s.	n.s.	n.s.
PE-P	1.313	0.243	0.526	0.136	0.916	0.166	<0.0001	0.0064	0.0078
PG	6.198	1.495	12.973	4.579	3.582	1.639	0.0061	0.0003	n.s.
PI	41.717	7.223	39.157	8.012	44.678	9.599	n.s.	n.s.	n.s.
PS	11.296	1.444	1.200	0.335	8.494	1.630	<0.0001	<0.0001	0.0066
SM	8.185	0.619	3.090	0.662	5.834	1.642	<0.0001	0.0020	0.0092
TAG	17.082	1.565	7.313	0.964	53.924	11.260	n.s.	<0.0001	<0.0001

DC, differential centrifugation; UC, ultracentrifugation; MACS, magnetic bead-assisted isolation; CE, cholesteryl ester; CER, ceramide; CL, cardiolipin; DAG, diacylglycerol; FFA, free fatty acids; HexCER, hexosyl-ceramide; LPC, lyso-phosphatidylcholine; LPE, lyso-phosphatidylethanolamine; LPI, lyso-phosphatidylinositol; PC, phosphatidylcholine; PE, phosphatidylethanolamine; PE-P, phosphatidylethanolamine plasmalogen; PG, phosphatidylglycerol; PI, phosphatidylinositol; PS, phosphatidylserine; SM, sphingomyelin; TAG, triacylglycerol. n.s., not significant. Values are means of at least 5 purifications.
