# Supplementary material for: Purity matters: A workflow for the valid high-resolution lipid profiling of mitochondria from cell culture samples
Source: Sci Rep. 2016 Feb 19;6:21107. doi: 10.1038/srep21107 (PMC4759577; doi:10.1038/srep21107)
Supplement: Supplementary Table 1 [file srep21107-s1.doc]

## Purity matters: A workflow for the valid high-resolution lipid profiling of mitochondria from cell culture samples

Lisa Kappler^1,+^, Jia Li^2,+^, Hans-Ulrich Häring^1,3,4^, Cora Weigert^1,3,4^, Rainer Lehmann^1,3,4^, Guowang Xu^2^,*, Miriam Hoene^1^,*

^1^Division of Clinical Chemistry and Pathobiochemistry, Department of Diagnostic Laboratory Medicine, University Hospital Tuebingen, Tuebingen, Germany

^2^Key Laboratory of Separation Science for Analytical Chemistry, Dalian Institute of Chemical Physics, Chinese Academy of Sciences, Dalian, China

^3^Department of Molecular Diabetology, Institute for Diabetes Research and Metabolic Diseases of the Helmholtz Centre Munich at the University of Tuebingen, Tuebingen, Germany

^4^German Center for Diabetes Research (DZD), Tuebingen, Germany

^+^Lisa Kappler and Jia Li contributed equally to this study.

*Corresponding Authors.

**SUPPLEMENT**

Supplementary Figure 1: Correlation of the sum of cardiolipins (CL) with the content of Mitochondrial ATP synthase 5 (ATP5) protein as determined by western blot analysis (r^2^= 0.79, p<0.0001).
