# Supplementary material for: Purity matters: A workflow for the valid high-resolution lipid profiling of mitochondria from cell culture samples
Source: Sci Rep. 2016 Feb 19;6:21107. doi: 10.1038/srep21107 (PMC4759577; doi:10.1038/srep21107)
Supplement: Supplementary Information [file srep21107-s2.pdf]

Purity matters: A workflow for the valid high-resolution lipid profiling of mitochondria from cell culture samples

Lisa Kappler[1,+], Jia Li[2,+], Hans-Ulrich Häring[1,3,4], Cora Weigert[1,3,4], Rainer Lehmann[1,3,4], Guowang Xu[2,*], Miriam Hoene[1,*]

[1]Division of Clinical Chemistry and Pathobiochemistry, Department of Diagnostic Laboratory Medicine, University Hospital Tuebingen, Tuebingen, Germany
[2]Key Laboratory of Separation Science for Analytical Chemistry, Dalian Institute of Chemical Physics, Chinese Academy of Sciences, Dalian, China
[3]Department of Molecular Diabetology, Institute for Diabetes Research and Metabolic Diseases of the Helmholtz Centre Munich at the University of Tuebingen, Tuebingen, Germany
[4]German Center for Diabetes Research (DZD), Tuebingen, Germany
[+]Lisa Kappler and Jia Li contributed equally to this study.
*Corresponding Authors.

SUPPLEMENT

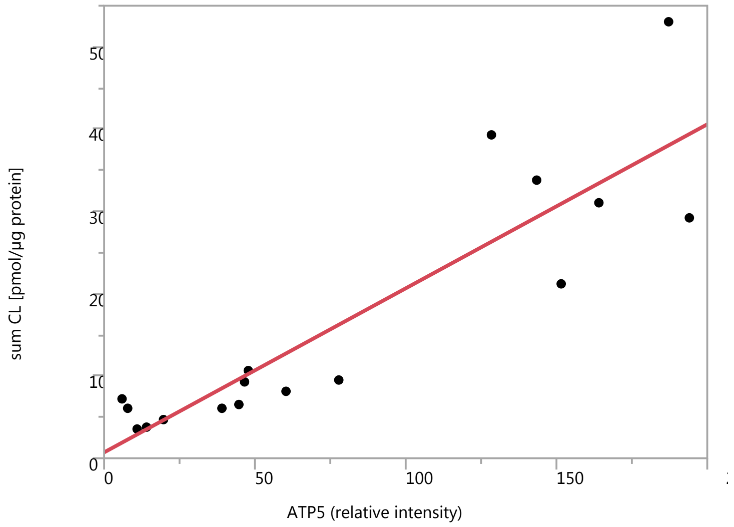

Supplementary Figure 1: Correlation of the sum of cardiolipins (CL) with the content of Mitochondrial ATP synthase 5 (ATP5) protein as determined by western blot analysis (r^2= 0.79, p<0.0001).
